# Near-Infrared Fluorescence Imaging With Indocyanine Green to Predict Clinical Outcome After Revascularization in Lower Extremity Arterial Disease

**DOI:** 10.1177/00033197231186096

**Published:** 2023-06-26

**Authors:** Floris P. Tange, Pim van den Hoven, Jan van Schaik, Abbey Schepers, Koen E.A. van der Bogt, Catharina S. P. van Rijswijk, Hein Putter, Alexander L. Vahrmeijer, Jaap F. Hamming, Joost R. van der Vorst

**Affiliations:** 1 Department of Surgery, 4501 Leiden University Medical Center, The Netherlands; 2 Department of Interventional Radiology, 4501 Leiden University Medical Center, The Netherlands; 3 Department of Medical Statistics, 4501 Leiden University Medical Center, The Netherlands

**Keywords:** lower extremity arterial disease, near-infrared fluorescence, indocyanine green, revascularization, clinical outcome

## Abstract

Contemporary quality control methods are often insufficient in predicting clinical outcomes after revascularization in lower extremity arterial disease (LEAD) patients. This study evaluates the potential of near-infrared fluorescence imaging with indocyanine green to predict the clinical outcome following revascularization. Near-infrared fluorescence imaging was performed before and within 5 days following the revascularization procedure. Clinical improvement was defined as substantial improvement of pain free walking distance, reduction of rest- and/or nocturnal pain, or tendency toward wound healing. Time-intensity curves and 8 perfusion parameters were extracted from the dorsum of the treated foot. The quantified postinterventional perfusion improvement was compared within the clinical outcome groups. Successful near-infrared fluorescence imaging was performed in 72 patients (76 limbs, 52.6% claudication, 47.4% chronic limb-threatening ischemia) including 40 endovascular- and 36 surgical/hybrid revascularizations. Clinical improvement was observed in 61 patients. All perfusion parameters showed a significant postinterventional difference in the clinical improvement group (*P*-values <.001), while no significant differences were seen in the group without clinical improvement (*P*-values .168–.929). Four parameters demonstrated significant differences in percentage improvement comparing the outcome groups (*P*-values within .002–.006). Near-infrared fluorescence imaging has promising additional value besides clinical parameters for predicting the clinical outcome of revascularized LEAD patients.

## Introduction

Lower extremity arterial disease (LEAD) affects over 230 million people worldwide and the burden of this disease is increasing.^1^ Besides medical treatment and supervised walking therapy, improvement of peripheral perfusion through revascularization is frequently performed in LEAD patients.^2^ However, despite successful revascularization, reintervention rates remain high, especially in patients suffering from chronic limb-threatening ischemia (CLTI).^3,4^ Presumably, a substantial amount of these intervention rates can be attributed to technical failure of the initial revascularization procedure, for example, stent or bypass occlusion.^4,5^ However, the lack of reliable (intraoperative) information about improvement of tissue perfusion at skin level could also contribute to the likelihood of undergoing successive interventions.

Currently, several modalities are used to measure the effect of a revascularization procedure. Ankle-brachial index (ABI), toe pressure (TP) measurement, and radiological imaging modalities are used to assess the postinterventional peripheral perfusion status; however, they only provide macrovascular information.^6^ The ABI is known to be unreliable in patients with diabetes mellitus and chronic kidney disease due to medial calcinosis and incompressible vessels.^6,7^ TP measurements can be more reliable in these patients, yet variable diagnostic accuracy has been described besides impractical measurement in toe amputation or ulceration.^6,8^ Additional imaging techniques consist of computed tomography angiography (CTA), magnetic resonance angiography (MRA), digital subtraction angiography (DSA), and duplex ultrasound (DUS). The added value of these tools complementary to clinical judgment has been proven, though objectivation of regional microvascular perfusion status can be of great added value as well, particularly in patients with ischemic wounds.^6,9^ This is underlined by current evidence that angiosome-guided revascularization might improve outcome in this subgroup of LEAD patients.^10^ Furthermore, the risk of developing contrast-induced nephropathy is a known limitation of some conventional imaging techniques.^11-13^

Transcutaneous oximetry (TcP02) measures regional tissue oxygen pressure and is mainly used to predict the likelihood of wound healing.^6,14^ Other experimental microvascular imaging techniques, including laser doppler flowmetry and hyperspectral imaging are emerging, although evidence on the ability to predict interventional effectiveness is still limited.^6,14-16^ Near-infrared (NIR) fluorescence imaging with indocyanine green (ICG) is a promising technique able to assess regional microvascular tissue perfusion and has already proven its value in various surgical disciplines.^17,18^ This technique uses the safe fluorescent coloring agent ICG to visualize blood flow. ICG emits a fluorescence signal after absorbing NIR light from a laser or LED source, which can be visualized by a NIR camera sensor creating real-time perfusion images.^19^ The usability of quantifying changes in the ICG NIR fluorescence signal has already been evaluated as a seemingly promising quality control method after revascularization.^9,20-22^ A potentially beneficial consecutive application is to correlate these quantitative perfusion changes to clinical outcome which could facilitate a more rapid interventional evaluation. In a study by Colvard et al., NIR fluorescence imaging was performed in LEAD patients undergoing revascularization, which demonstrated a significant improvement in quantitative perfusion for patients with clinical improvement.^23^ However, standardization of quantification continues to be a challenge. Therefore, this study aims to address the value of quantifying changes in standardized perfusion measurements for the prediction of clinical outcome following revascularization in LEAD patients.

## Methods

This prospective cohort study was approved by the Medical Research and Ethics Committee of the Leiden University Medical Center. The NIR fluorescence imaging procedure and in- and exclusion criteria were in accordance with a previously described protocol.^9^ All patients who underwent a technically successful revascularization and successful pre- and postinterventional ICG NIR fluorescence imaging in a single academic hospital between July 2019 and December 2021 were included in this study. Patients which were included before implementation of ICG NIR fluorescence imaging as standard of care provided written informed consent. All patients provided permission for data usage. Outpatient clinic follow-up was performed within 6 weeks following the revascularization procedure according to protocol. Patients with either bypass or stent occlusion on postoperative duplex ultrasound imaging were defined as technical failure and excluded from analyses. Information concerning clinical outcome at the moment of follow-up was obtained from the patient records.

Patients were divided in two groups depending on their clinical outcome: clinical improvement or no clinical improvement. Clinical improvement was defined as a substantial improvement of pain free walking distance, reduction or disappearance of rest or nocturnal pain, or tendency towards wound or ulcer healing assessed by the treating physician. Pre- and postinterventional TP and ABI measurements were performed, if possible.

### Measurement Setup

The Quest Spectrum Platform ® (Quest Medical Imaging, Middenmeer, the Netherlands) was used for the ICG NIR fluorescence imaging. This system uses a visible light engine and an NIR light source (700-820 nm). Patients were administered an intravenous bolus administration of .1 mg/kg indocyanine green (Verdye, Diagnostic Green GmbH, Aschheim-Dornach, Germany) in an antecubital vein.

Following administration, the camera registered the near-infrared fluorescence intensity over time in both feet for 10 min. Fluorescence measurements were performed by a senior clinical researcher within 1 day prior to the intervention and within 5 days after the intervention.

### Quantification

Post-process quantification of the fluorescence signal was performed using the Quest Research Framework® (Quest Medical Imaging, Middenmeer, the Netherlands). This software program generates absolute- and normalized time-intensity curves in a chosen region of interest (ROI), which for this study was set at the dorsum of the foot on the intervention side. A build-in motion tracker adjusted the image for foot movement during the ICG NIR fluorescence measurement. Normalization of time-intensity curves was performed according to a previously described protocol.^24^ Multiple inflow and outflow parameters were extracted from the time-intensity curves, outlined in Supplemental Figure 1. The difference in pre- and postinterventional perfusion parameters were described as percentage changes. Percentage improvement of perfusion parameters was defined as an increase in inflow parameter values and a decrease in outflow parameter values.

### Statistical Analyses

Statistical analyses were conducted using IBM SPSS Statistics 25 (IBM Corp. Released 2017. IBM SPSS Statistics for Windows, version 25.0. Armonk, NY, USA: IBM Corp.). The percentage improvement of perfusion parameters were compared between the outcome groups with a Mann–Whitney U test. Pre- and postinterventional perfusion parameters and conventional ABI and TP were compared using the Wilcoxon signed rank test. Statistical outcomes were significant when the *P*-value was <.05. Receiver operating characteristics (ROC) curves were plotted depicting the predictive value of perfusion parameters for clinical improvement.

## Results

### Patient Characteristics

Successful pre- and postinterventional ICG NIR fluorescence imaging was performed in 72 patients (Table 1). No adverse events were observed, including no decline in kidney function (eGFR) after ICG administration. The clinical improvement group consisted of 61 patients (65 limbs). Within this group, 37 limbs were classified with claudication (56.9%) and 28 limbs were classified with CLTI (56.9%), of which 12 limbs had wound(s) (42.9%). Surgical revascularization was performed in 30 limbs, endovascular revascularization in 30 limbs and a hybrid procedure in five limbs (46.2, 46.2%, and 7.6%, respectively). Eighteen limbs were revascularized at an aortoiliac level (27.7%), 41 limbs at a femoro-popliteal level (63.1%), and six limbs (9.2%) at a crural level. The mean follow-up was 38.3 (±20.6) days. No clinical improvement was reported in 11 patients (11 limbs). The characteristics of patients without clinical improvement are shown in Supplementary Table 1. Three limbs were classified with claudication (27.3%) and eight limbs were classified with CLTI (72.7%), of which seven had wound(s) (87.5). Ten limbs were treated endovascularly (90.9%) and one limb was treated surgically (9.1%). Three limbs (27.2%) were revascularized on an aortoiliac level, four limbs (36.4%) were treated on femoral/popliteal level, and four limbs (36.4%) were treated on a crural level. The mean follow-up was 27.3 (±17.1) days.

**Table 1.** Patient characteristics.^a^

	Clinical improvement (n = 61, limbs = 65)	No clinical improvement (n = 11, limbs = 11)	Total (n = 72, limbs = 76)
Age (years)	70.1 ± 8.0	73.3 ± 7.6	70.6 ± 8.0
Body mass index	26.1 ± 4.3	27.4 ± 4.8	26.3 ± 4.4
Female─n(%)	26 (42.6)	6 (54.5)	32 (44.4)
Hypertension─n(%)	45 (73.8)	9 (81.1)	54 (75)
Diabetes─n(%)	21 (34.4)	6 (54.5)	27 (37.5)
Active smoking─n(%)	11 (18)	4 (36.4)	15 (20.8)
History of smoking─n(%)	56 (91.8)	7 (63.6)	63 (87.5)
- Missing	1 (1.6)	0	1 (1.4)
Renal insufficiency─n(%)	3 (4.9)	2 (18.2)	5 (6.9)
Baseline ABI	.7 ± 0.3	.6 ± 0.3	.7 ± .3^b^
Baseline TP (mmHg)	67.5 ± 30.4	46.7 ± 22.9	66.0 ± 30.2^c^
Mean follow-up (days)	38.3 ± 20.6	27.3 ± 17.1	36.9 ± 20.2
Clinical presentation─n(%)			
Claudication	37 (56.9)	3 (27.3)	40 (52.6)
CLTI	28 (43.1)	8 (72.7)	36 (47.4)
Wound(s)	12 (42.9)	7 (87.5)	19 (52.8)
Type of revascularization─n(%)			
Endovascular	30 (46.2)	10 (90.9)	40 (52.6)
Surgical	30 (46.2)	1 (9.1)	31 (40.8)
Hybrid	5 (7.6)	0	5 (6.6)
Level of revascularization─n(%)			
Aortoiliac	18 (27.7)	3 (27.2)	21 (27.6)
Femoral/popliteal	41 (63.1)	4 (36.4)	45 (59.2)
Crural	6 (9.2)	4 (36.4)	10 (13.2)

Abbreviations: ABI: ankle-brachial index; TP: toe pressure; CLTI: chronic limb-threatening ischemia.

^a^Plus–minus values are means ± SD.

^b^Obtained in n = 56 limbs.

^c^Obtained in n = 42 limbs.

### Outcomes

**Figure 1.**
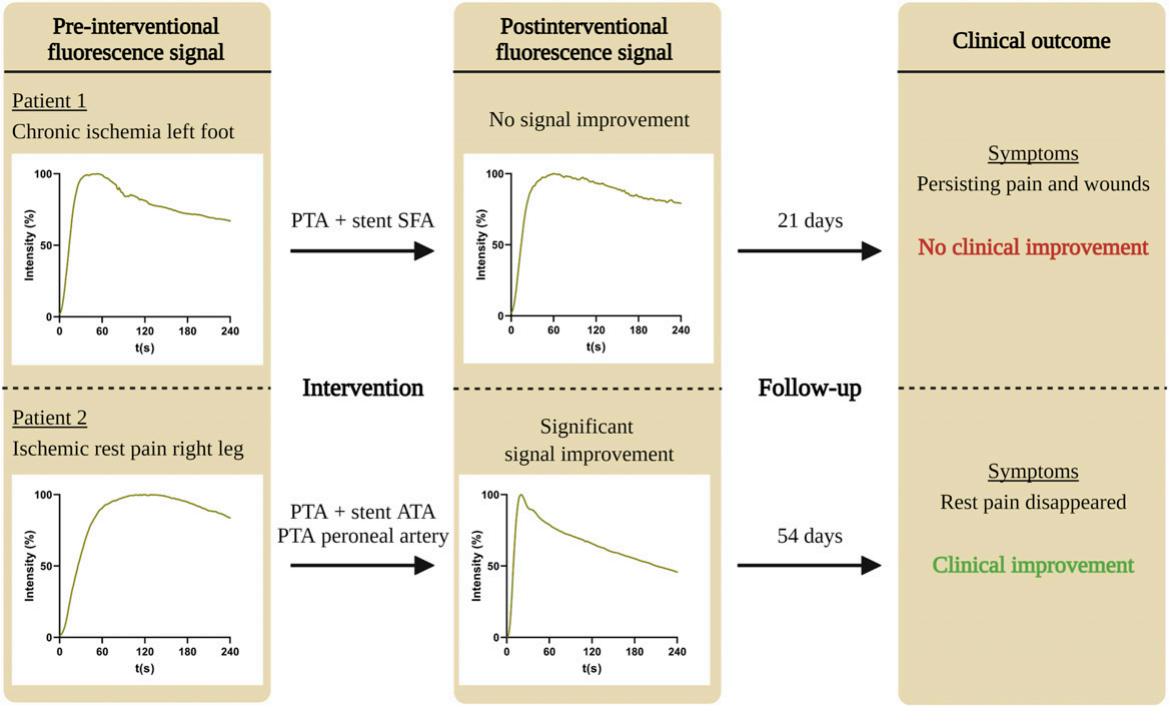
Flowchart of two patient cases with clinical improvement (*upper panel*) and without clinical improvement (*lower panel*), depicting the pre-interventional time-intensity curve, the type of intervention, postinterventional time-intensity curve, and clinical outcome. Abbreviations: PTA: percutaneous transluminal angioplasty; SFA: superficial femoral artery; ATA: anterior tibial artery.

Pre- and postinterventional results of the ICG NIR fluorescence measurements are displayed in Table 2. A flowchart of two included patient cases with- and without clinical improvement and corresponding pre- and postinterventional fluorescence signal is displayed in Figure 1. All 8 perfusion parameters in the clinical improvement group displayed a significant postinterventional difference with *P*-values <.001.

**Table 2.** Pre- and postinterventional ICG NIR fluorescence parameters.

	Clinical improvement			No clinical improvement		
	Pre	Post	***P***	Pre	Post	***P***
Parameters	Median (quartiles)	Median (quartiles)		Median (quartiles)	Median (quartiles)	
Imax	33.7 (23.3/44.0)	46.8 (32.5/59.7)	<.001	46.6 (39.4/66.1)	55.8 (43.3/80.1)	.328
Absolute slope	1.0 (.5/2.0)	2.4 (1.3/4.0)	<.001	2.6 (1.1/5.3)	3.4 (1.1/4.0)	.790
Ingress rate	.3 (.2/.8)	1.0 (.4/2.0)	<.001	1.1 (.4/2.4)	1.4 (.4/2.1)	.929
Normalized slope	3.3 (2.2/4.4)	5.5 (3.6/7.4)	<.001	5.2 (2.8/8.2)	6.1 (2.5/6.7)	.534
Tmax	88.5 (50.0/151.5)	43.5 (24.7/81.9)	<.001	49.3 (18.9/79.8)	52.2 (21.8/143.6)	.168
AUC egress 60	96.8 (94.1/97.8)	92.7 (86.7/96.7)	<.001	92.2 (76.7/97.5)	92.9 (81.3/97.1)	.859
AUC egress 120	92.2 (85.7/94.6)	885.6 (76.0/91.0)	<.001	84.7 (67.4/93.5)	81.5 (70.4/91.6)	.575
AUC egress 180	86.3 (77.2/90.3)	78.2 (69.0/86.0)	<.001	79.3 (60.6/88.3)	74.5 (63.4/87.0)	.721

Abbreviations: Imax: maximum intensity; Tmax: time to max; AUC: area under the curve.

**Figure 2.**
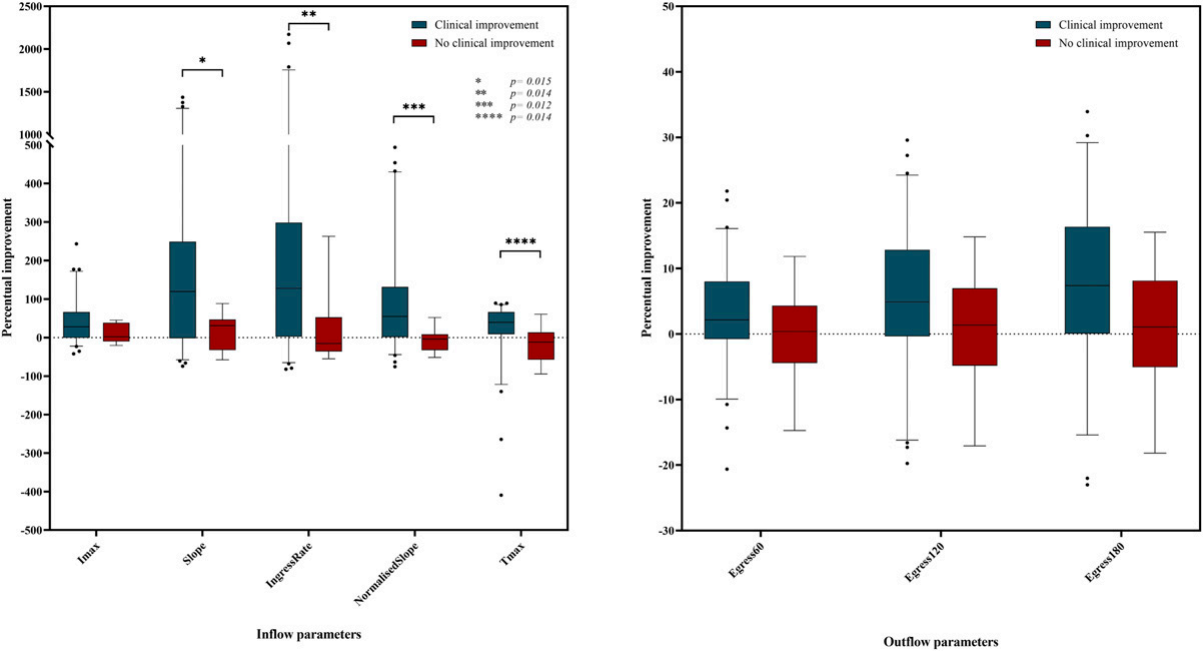
Boxplots of percentual inflow- (*left panel*) and outflow (*right panel*) parameter improvement for the clinical improvement and no clinical improvement group. Abbreviations: Imax: maximum intensity; Slope: absolute slope ingress; Tmax: time to maximum intensity; Egress: area under the curve egress.

No significant differences were observed for the same parameters in the group without clinical improvement (*P*-values between .168 and .929). The percentage improvement of all perfusion parameters in both groups are displayed in Table 3 and Figure 2. Significant differences in percentual improvement comparing the outcome groups were observed in four inflow parameters (absolute slope: *P* = .006, ingress rate: *P* = .004, normalized slope: *P* = .003, and time to maximum intensity (Tmax): *P* = .002). Of these parameters, the normalized slope, ingress rate, and Tmax showed positive median percentage improvements in the clinical improvement group, while negative median percentage improvements were seen in group without clinical improvement. Outflow parameters and the maximum intensity (Imax) displayed no significant differences in percentage improvement between the outcome groups.

**Table 3.** Percentual Improvement Perfusion Parameters After Revascularization.

Parameters	Clinical improvement	No clinical improvement	***P***
	Median percentage (quartiles)	Median percentage (quartiles)	
Imax	27.9 (−.6/66.2)	2.1 (−10.1/38.4)	.142
Absolute slope	119.8 (−1.9/249.3)	31.0 (−32.5/47.1)	.006
Ingress rate	127.8 (2.5/298.5)	−15.3 (−36.1/52.6)	.004
Normalized slope	55.0 (1.3/131.6)	−4.1 (−32.8/8.5)	.003
Tmax	39.5 (8.9/66.3)	−11.8 (−57.1/13.8)	.002
AUC egress 60	2.2 (−.8/8.0)	.4 (−4.4/4.3)	.235
AUC egress 120	4.9 (−.3/12.8)	1.4 (−4.8/7.0)	.186
AUC egress 180	7.4 (.0/16.4)	1.1 (−5.0/8.1)	.104

Abbreviations: Imax: maximum intensity; Tmax: time to max; AUC: area under the curve.

**Figure 3.**
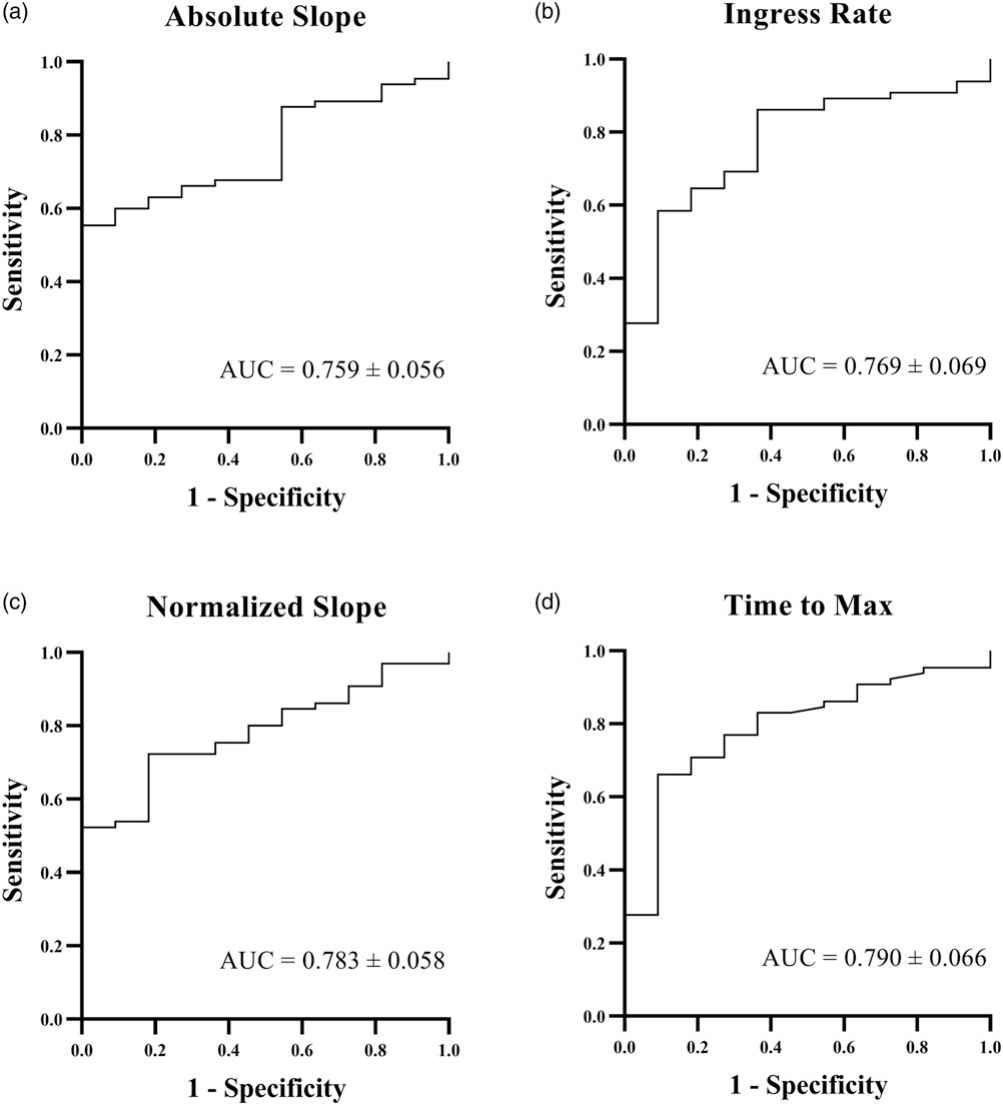
Receiver operating characteristic (ROC) curves for the percentual improvement of the absolute slope (A, AUC: .759 ± .056), ingress rate (B, AUC: .769 ± .069), normalized slope (C, AUC: .783 ± .058), and time to max (D, AUC: .790 ± .066) for predicting clinical improvement.

A significant postinterventional increase in ABI and TP was observed within the clinical improvement group, while no significant difference in ABI and TP was seen in the group without clinical improvement (*P* = <.001, *P* = <.001; *P* = .398, *P* = .655, respectively). Receiver operating characteristics (ROCs) curves of the four significantly differing inflow parameters are presented in Figure 3. The area under the ROC curves was similar in all four parameters: .759 for the absolute slope (Figure 3A), .769 for ingress rate (Figure 3B), .783 for normalized slope (Figure 3C), and .790 for Tmax (Figure 3D).

## Discussion

This study evaluates the potential of ICG NIR fluorescence imaging as an additional tool for the prediction of clinical outcome following lower extremity revascularization. A significant percentage improvement was seen in several inflow parameters for patients with clinical improvement. Moreover, no significant pre- and postinterventional improvement was seen for the same parameters in patients without clinical improvement. These results demonstrate the promising usability of quantitative ICG NIR fluorescence imaging for predicting clinical outcome after revascularization and imply a strong recommendation for further appraisal of this application considering the potentially clinically relevant added value. Direct intraoperative or postinterventional assessment of perfusion alterations using this technique could provide guidance in deciding the extensiveness of a revascularization and in personalizing follow-up and treatment strategy.

Several studies already showed the usefulness of quantitative ICG NIR fluorescence imaging as a quality control measure following revascularization.^9,20-22^ Igari et al. showed the ability of multiple perfusion parameters to demonstrate changes in peripheral perfusion after revascularization.^21^ Although quantitative ICG NIR fluorescence imaging can be used to quantify postinterventional changes in peripheral perfusion, evidence on predicting the clinical outcome is limited. In a study performed by Colvard et al, ICG NIR fluorescence measurements before and after revascularization demonstrated significant improvement of perfusion parameters in patients with clinical improvement, while no significant changes were observed for patients without clinical improvement.^23^ Unlike the present study, Colvard et al. analyzed the plantar surface of the foot and only three absolute intensity-related perfusion parameters were analyzed.

Predicting the extent of successful outcome following different intervention types and in various patient groups remains a substantial challenge. The present study cohort consisted of claudication and CLTI patients, in whom the definition of clinical improvement is subjective and varies considerably. Besides, foot-related problems of CLTI patients are directly evaluated by perfusion assessment with ICG NIR fluorescence, while claudicants suffer from lower leg muscle malperfusion which is therefore more indirectly evaluated by detecting perfusion alterations of the foot. Furthermore, endovascular revascularized patients shown higher reintervention rates compared with surgical treatment, therefore creating more room for improvement in this group.^4^ A large prospective trial (n = 452) demonstrated long-term reintervention rates after a percutaneous transluminal angioplasty (PTA) to be 25.9% while 18.3% after bypass surgery in CLTI patients.^4^ The present study examined relatively short-term clinical outcomes defined as symptom- or wound improvement. However, LEAD is a chronic disease which makes the prediction of long-term clinical outcome such as reintervention rate and amputation free survival relevant as well, especially in advanced CLTI.

Several large studies tried to create a prognostic scoring system for these clinical outcomes in CLTI, resulting in the wound, ischemia, and foot infection (WIfI) classification for instance.^25^ A variety of clinical parameters, as well as outcomes of diagnostic tools were included as risk elements, emphasizing the broad variety of components influencing the clinical outcome.^26^ These diagnostic tools include postintervention ABI and TP changes which were shown to be of added value and often sufficient in predicting clinical outcome, yet both tools are ineffective in several common circumstances.^6-8,27,28^ Furthermore, TcP02 is able to predict the likelihood of wound- and ulcer healing in CLTI patients; however, research shows that optimal postinterventional cutaneous oxygenation levels can take up to 4 weeks to be reached.^6,14,29^ ICG NIR fluorescence can be more broadly applied with the only requirement that a patient has no contra-indication for ICG.

In this explorative study, quantitative ICG NIR fluorescence has proved its potential for predicting clinical outcome after revascularization, though performing an accurate predictive value analysis along with cut-off points for percentual parameter improvement was not feasible due to the small sample size. The heterogeneity of intervention types, level of revascularizations and clinical presentations (claudication/CLTI) limits this study and are recommended to be included in sub-analysis when accurately assessing the predictive value of this technique is a larger cohort. Furthermore, the assessment of wound healing by the treating physician was subjective in this study and could be improved by using more objective wound healing measures.

Early detection of perfusion alterations through quantitative ICG NIR fluorescence could lead to a more accurate prediction of postinterventional clinical outcome and if necessary to a more rapid transition towards the following interventional treatment step instead of awaiting symptom- or wound improvement. This would allow for better interventional decision making and more personalized follow-up. Additionally, intraoperative assessment of perfusion alterations in for instance endovascular revascularizations could potentially be used to immediately decide whether the intervention should be extended. In these circumstances, intra-arterial administration of ICG might be beneficial relative to intravenous administration for immediate perfusion assessment.^30^ These applications are recommended to be investigated in future studies for providing reliable guidance in revascularization strategy.

## Conclusions

The present study shows a significant improvement of perfusion measured with ICG NIR fluorescence imaging in patients with clinical improvement following revascularization. Interestingly, no significant improvement was seen in patients without clinical improvement. This study underlines the potential of ICG NIR fluorescence in addition to clinical examination for predicting the clinical outcome after revascularization. These findings should be confirmed by future studies, additionally evaluating possible cut-off values for reliable inflow parameters.

## Supplemental Material

Supplemental Material - Near-Infrared Fluorescence Imaging With Indocyanine Green to Predict Clinical Outcome After Revascularization in Lower Extremity Arterial Disease Supplemental Material for Near-Infrared Fluorescence Imaging With Indocyanine Green to Predict Clinical Outcome After Revascularization in Lower Extremity Arterial Disease by Floris P. Tange, Pim van den Hoven, Jan van Schaik, Abbey Schepers, Koen van der Bogt, Catharina S. P. van Rijswijk, Hein Putter, Alexander L. Vahrmeijer, Jaap F. Hamming, and Joost R. van der Vorst in Angiology
