# Supplementary material for: Near-Infrared Fluorescence Imaging With Indocyanine Green to Predict Clinical Outcome After Revascularization in Lower Extremity Arterial Disease
Source: Angiology. 2023 Jun 26;75(9):884–92. doi: 10.1177/00033197231186096 (PMC11375904; doi:10.1177/00033197231186096)
Supplement: Supplemental Material - Near-Infrared Fluorescence Imaging With Indocyanine Green to Predict Clinical Outcome After Revascularization in Lower Extremity Arterial Disease [file sj-pdf-1-ang-10.1177_00033197231186096.pdf]

Supplementary Table I. Characteristics of patients without clinical improvement

**Case**	Gender	Age	Diabetes	Renal insufficiency	Clinical presentation	Wound(s)	Intervention	Level of revascularization	Follow-up (days)	Clinical outcome
1	F	67	No	No	Claudication	No	PTA + stent	SFA	21	Persisting claudication
2	M	82	Yes	No	CLTI	Yes	PTA	PTA + ATA	40	Progression wound necrosis
3	M	85	No	Yes	CLTI	Yes	PTA	PTA	45	Persisting wound
4	M	73	No	Yes	CLTI	Yes	PTA + stent	SFA	21	Persisting wounds and pain
5	M	70	No	No	Claudication	No	PTA	CIA	14	Increased claudication
6	M	57	Yes	No	CLTI	Yes	PTA	DPA + ATA	62	Persisting wounds and increased pain
7	F	76	Yes	No	CLTI	Yes	PTA	Tibioperoneal trunk	37	Persisting wounds and pain
8	F	77	Yes	No	CLTI	No	PTA + stent	CIA + EIA	6	Increased ischemia and pain
9	F	71	No	No	Claudication	No	PTA + stent	EIA	42	Persisting claudication
10	F	71	Yes	No	CLTI	Yes	PTA + stent	PA + SFA	22	Additional wound formation
11	F	77	Yes	No	CLTI	Yes	TEA	CFA + DFA	12	Increased ischemia and pain

Abbreviations: F, Female, M, Male, CLTI, Critical Limb-Threatening Ischemia, PTA (intervention), Percutaneous Transluminal Angioplasty, SFA, Superficial Femoral Artery, PTA (level of revascularization), Posterior Tibial Artery, ATA, Anterior Tibial Artery, CIA, Common Iliac Artery, Dorsalis Pedis Artery, EIA, External Iliac Artery, PA, Peroneal Artery, CFA, Common Femoral Artery, DFA, Deep Femoral Artery

For Peer Review

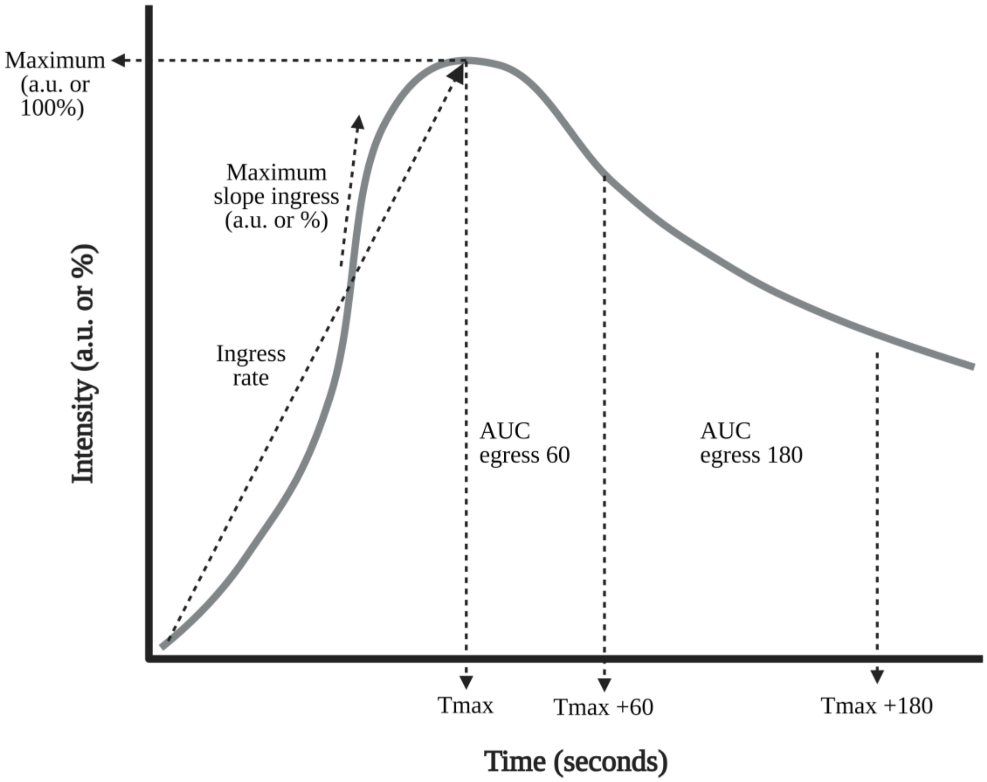

Time-intensity curve with extracted parameters. Abbreviations: a.u., arbitrary units; AUC, Area Under the Curve; Tmax, Time to Max.

188x149mm (600 x 600 DPI)
